# Myostatin Activates the Ubiquitin-Proteasome and Autophagy-Lysosome Systems Contributing to Muscle Wasting in Chronic Kidney Disease

**DOI:** 10.1155/2015/684965

**Published:** 2015-09-13

**Authors:** Dong-Tao Wang, Ya-Jun Yang, Ren-Hua Huang, Zhi-Hua Zhang, Xin Lin

**Affiliations:** ^1^Department of Nephrology, Ruikang Hospital, Guangxi University of Chinese Medicine, Nanning 530011, China; ^2^Department of Pharmacology, Guangdong Key Laboratory for R&D of Natural Drug, Guangdong Medical College, Zhanjiang 524023, China; ^3^Department of Traditional Chinese Medicine, General Hospital of Guangzhou Military Command of PLA, Guangzhou 510010, China; ^4^Division of Nephrology, Nanfang Hospital, Southern Medical University, Guangzhou 510280, China

## Abstract

Our evidence demonstrated that CKD upregulated the expression of myostatin, TNF-*α*, and p-IkBa and downregulated the phosphorylation of PI3K, Akt, and FoxO3a, which were also associated with protein degradation and muscle atrophy. The autophagosome formation and protein expression of autophagy-related genes were increased in muscle of CKD rats. The mRNA level and protein expression of MAFbx and MuRF-1 were also upregulated in CKD rats, as well as proteasome activity of 26S. Moreover, activation of myostatin elicited by TNF-*α* induces C2C12 myotube atrophy via upregulating the expression of autophagy-related genes, including MAFbx and MuRF1 and proteasome subunits. Inactivation of FoxO3a triggered by PI3K inhibitor LY294002 prevented the myostatin-induced increase of expression of MuRF1, MAFbx, and LC3-II protein in C2C12 myotubes. The findings were further consolidated by using siRNA interference and overexpression of myostatin. Additionally, expression of myostatin was activated by TNF-*α* via a NF-*κ*B dependent pathway in C2C12 myotubes, while inhibition of NF-*κ*B activity suppressed myostatin and improved myotube atrophy. Collectively, myostatin mediated CKD-induced muscle catabolism via coordinate activation of the autophagy and the ubiquitin-proteasome systems.

## 1. Introduction

Chronic kidney disease (CKD) is associated with muscle wasting, which directly correlates with mortality and morbidity [1]. Several conditions associated with CKD, such as acidosis, insulin resistance, inflammation, and increased glucocorticoid and angiotensin II production can promote muscle wasting through an increase in protein degradation and/or a decrease in protein synthesis [2]. However, it is increasingly evident that CKD per se predisposes to muscle wasting [3, 4]. Recent advances obtained in experimental CKD have consistently increased our understanding of intracellular pathways producing loss of muscle mass. Inflammation increases protein degradation through an upregulation of the ATP-dependent, ubiquitin-requiring pathway [5]. In addition, apoptosis regulated muscle protein degradation through activation of caspase-3 to cleave the myofibril into actin, myosin, troponin, and tropomyosin [6].

The principal mechanism causing muscle atrophy in CKD involves activation of the ubiquitin-proteasome system (UPS) [2]. In the UPS, proteins are tagged through covalent attachment of a chain of ubiquitin molecules to their proteolytic substrates for degradation by the 26S proteasome. The 26S proteasome is composed of one 20S core proteasome and two 19S complexes. The 19S complex can also catalyze the unfolding of substrate proteins and facilitate transport of the unfolded proteins into the 20S core proteasome, which include the chymotrypsin- and trypsin-like activities and several proteasome subunits (PSMA, Rpt, and Rpn) [7]. Meanwhile, it has been reported that the chymotrypsin- and trypsin-like activities and the proteasome subunits are elevated in compression-induced muscle atrophy [8]. The two novel muscle-specific ubiquitin ligases, MAFbx and MuRF1, that are upregulated in several models of muscle atrophy and promote muscle protein degradation by the UPS [9, 10]. In fact, knockout mice for either MAFbx or MuRF1 are partially resistant to muscle atrophy caused by denervation [9]. Importantly, recent studies suggest that myosin heavy chain (MHC) is ubiquitinated and degraded by MuRF1 both in vitro and in vivo [11, 12]. Thus, up to now, these two genes are actually the best markers for muscle atrophy and could be considered as master genes for muscle wasting.

Macroautophagy, hereafter referred to as autophagy, is the autophagy-lysosomal system (ALS) that is activated in catabolic conditions [13]. Autophagy is a highly conserved homeostatic mechanism that is used for the degradation and recycling, through the lysosomal machinery of bulk cytoplasm, long-lived proteins, and organelles [14]. Recent studies have demonstrated autophagy activation in skeletal muscle in a variety of conditions and diseases, such as denervation and fasting [15]. Indeed, autophagy may trigger the selective removal of specific organelles, such as mitochondria, via mitophagy. It must be noted that the same signal can theoretically cause cell and protein loss, as Akt can mediate the effects of FoxO3a on different events, such as autophagy and protein catabolism. Recently, it was reported that FoxO3a is necessary and sufficient for the induction of autophagy in skeletal muscle [16].

Myostatin, a member of the TGF-*β* family, is expressed and secreted predominantly by skeletal muscle and functions as a negative regulator of muscle growth. The potential importance of myostatin has been underscored by studies obtained in humans, in which muscle myostatin has been shown to increase with aging, in muscle atrophy and in chronic illnesses [17, 18]. Indeed, muscles from myostatin-null mice lead to dramatic increases in skeletal muscle mass due to muscle fiber hyperplasia and/or hypertrophy [19, 20]. In addition, natural inactivating mutations of the myostatin gene have been shown to be associated with double muscling in cattle [21–23]. Conversely, transgenic mice with muscle-specific overexpression of myostatin in skeletal muscle have lower muscle mass [24]. However, the downstream targets of the myostatin pathway and their role in protein synthesis as well as protein degradation are not well understood.

The NF-*κ*B transcription factors, which are expressed in skeletal muscle, appear to mediate the effect of inflammatory cytokines, in particular TNF-*α*, on muscle wasting. Nuclear factor-*κ*B activation has been shown to be required for protein loss in muscle wasting associated with chronic inflammation. In the inactive state, NF-*κ*B is sequestered in the cytoplasm by a family of inhibitory proteins called I*κ*B. In response to TNF-*α*, these proteins are phosphorylated by I*κ*B kinase (IKK) complexes, resulting in their ubiquitination and proteasomal degradation; this leads to translocation to the nucleus, binding of consensus sequence, and subsequent gene transcription [25].

In this study, we examined whether UPS and ALS can contribute to the development of muscle wasting. In addition, we studied how myostatin regulates muscle UPS and ALS responses. Moreover, we studied the role of several other factors, including PI3K/Akt/FoxO3a signaling pathway and inflammatory mediators, as potential predictors of muscle autophagy-related genes and myostatin expression. Our data show that myostatin regulates the UPS and ALS via the PI3K/Akt/FoxO3a signaling contributing to muscle wasting in CKD.

## 2. Materials and Methods

### 2.1. Reagents and Antibodies

Phosphatase inhibitor, protease inhibitor, and LY294002 were obtained from Roche (Indianapolis, IN), chicken anti-goat IgG-FITC, protein A/G Plus beads from Santa Cruz Biotechology (Santa Cruz, CA), RNAeasy and Plasmid Maxi Kit from Qiagen (Valencia, CA), the iScript cDNA Synthesis Kit from Bio-Rad (Hercules, CA), and Protein Block Serum Free and Antibody Diluent from Dako (Glostrup, Denmark). Vectastain Elite ABC Peroxidase kit and Imm PACT DAB peroxidase substrate were from (Vector Laboratories, Burlingame, CA, USA), TNF-*α* was from R&D Systems (Minneapolis, MN), QNZ, the NF-*κ*B inhibitor, was from Enzo Life Sciences (Ann Arbor, MI), and the myostatin siRNA and control siRNA were from Santa Cruz Biotechnology. The Nucleofector kit and GFP plasmid were from Lonza (Allendale, NJ). Myostatin plasmid cDNA was from Open Biosystems (Lafayette, CO), C2C12 mouse myoblasts were from The Type Culture Collection of the Chinese Academy of Sciences (Shanghai, China), and Dulbecco's modified Eagle's medium and fetal bovine serum were from Cellgro Mediatech (Manassas, VA). The antibodies against phospho-PI3K (Tyr 508), total PI3K, phospho-Akt (Ser 473), total Akt, phospho-FoxO3a (Ser 253), total FoxO3a, p-FoxO1, p-I*κ*Ba (Ser 32), Atg3, Atg7, Atg12, Beclin-1, and LC3-I/II were from Cell Signaling Technology (Beverly, MA), against myostatin was from Cell Applications (San Diego, CA), against MAFbx, MuRF1, PAMA4, Rpt5, Rpn11, glyceraldehydes 3-phosphate dehydrogenase (GAPDH), and TNF-R1 were from Santa Cruz Biotechnology.

### 2.2. CKD Model and Cell Culture

Male Sprague-Dawley rats obtained from Experimental Animal Center of Southern Medical University, with the certification number: SCXK (Yue) 2006-0015. Animal experiments were approved by the ethics committee of Southern Medical University. Anesthetized rats underwent subtotal nephrectomy in two stages as described previously [26]. Each animal in the nephrectomy group underwent 5/6 nephrectomy by ablation of two-thirds mass of the left kidneys and subsequent right unilateral nephrectomy after one week. In a sham-operated rat, a sham operation was performed. Each group included ten rats. Rat were anesthetized at the end of the study for 24 weeks; tibialis anterior (TA) and soleus and gastrocnemius muscles were obtained.

C2C12 cells were cultured and differentiated as described previously [27]. Differentiated myotubes were treated with a dose of TNF-*α* as indicated for 24 h.

### 2.3. Blood and Urine Examination

The 24 h urine samples were collected by using metabolism cages. Aortic blood obtained from anesthetized rats were used to measure serum creatinine (SCr); blood urea nitrogen (BUN) and serum albumin were measured using a commercial kit (Roche Diagnostics, Roche, Basel, Switzerland) and 24 h urinary protein excretion was measured with another commercial kit (Tonein-TPII, Ot-suka, Tokushima, Japan) according to the instructions of the manufacturers.

### 2.4. Histochemistry and Myofiber Cross-Sectional Area Measurements

After sacrificing the rats, TA muscles were fixed in paraformaldehyde and embedded in paraffin. The muscles were sectioned and stained with hematoxylin and eosin (H&E) in line with standards. Myofiber cross-sectional area was then determined in the way as previously reported [28]. Six sections of 50 contiguous myofibers were demarcated in each muscle so that an average of 300 fibers was obtained for fiber area measurement. With the aid of an image morphometry program (Image J 1.32 j, NIH, Bethesda, MD, USA), the borders were delineated with a calibrated pen by circling each fiber. Each fiber was further traced with a handheld mouse to pixel of *μ*m^2^ in area.

### 2.5. Protein Synthesis and Protein Degradation

Protein synthesis was measured in vitro in the soleus muscle using the incorporation of 14-C phenylalanine (phe) as previously described [29]. 14-C-Phe [0.05 *μ*Ci/mL] was obtained from China Institute of Atomic Energy (Beijing, China). Protein synthesis was calculated by dividing the protein-bound radioactivity by the specific activity of the free phenylalanine in the incubation medium and expressed as p moles of phe incorporated per milligram of protein per 2 h.

Protein degradation was measured using tyrosine release from isolated muscle as previously described by Rannels et al. [30]. Tyrosine was purchased from Sigma-Aldrich. The soleus muscle strips were preincubated for 30 min in Krebs Ringer buffer [NaCl 1.2 mmol/L; KCl 4.8 mmol/L; NaHCO_3_ 25 mmol/L; CaCl_2_ 2.5 mmol/L; KH_2_PO_4_ 1.2 mmol/L and MgSO_4_ 1.2 mmol/L; ph 7.4], supplemented with glucose [5.5 mmol/L], BSA [1.0 g/L], insulin [5 U/mL], and cyclohexamide [5 mmol/L], saturated with 95% O_2_/5% CO_2_ gas mixture. They were then transferred into fresh medium of the same composition and incubated for 2 h. At the end of the incubation, samples of the incubation medium were used for the assay of tyrosine by the procedure of Waalkes and Udenfriend [31].

### 2.6. Proteasome Activity Assay

Muscle cell lysates were prepared by homogenizing the gastrocnemius muscle in: 50 mM Tris, 1 mM EDTA, 5 mM MgCl_2_, 150 mM NaCl, and 1 mM DTT, pH 7.5. The samples were then centrifuged at 12,000 g for 10 min and the supernatants were collected. Proteasome activity of muscle homogenates (20 mg/sample) was measured with fluorescent substrates of Z-LLE-AMC (*β*1), Boc-LSTR-AMC (*β*2), and Suc-LLVY-AMC (*β*5) as previously described [32]. Assays were carried out in a total volume of 100 mL. The ATP-dependent 26S proteasome activities were measured in the presence of 50 mM Tris, 1 mM EDTA, 150 mM NaCl, 10 mM MgCl_2_, and 0.1 mM ATP, pH 7.5. The assay was conducted in the absence and presence of a specific proteasome inhibitor (40 mM Z-Pro-Nle-Asp-H for *β*1, 60 mM epoxomicin for *β*2, and 20 mM epoxomicin for *β*5) to determine proteasome-specific activity. Released AMC was measured using a Fluoroskan Ascent fluorometer (Thermo Electron) at an excitation wavelength of 390 nm and an emission wavelength of 460 nm.

### 2.7. Immunohistochemistry and Immunofluorescent Staining

Frozen tissue cross-sections were cut to a thickness of 10 *μ*m in a cryostat at −20°C. The sections were air dried at room temperature and fixed with 10% formalin solution. Background activity was minimized by blocking the section with 5% goat serum. After rinses, sections were incubated with anti-myostatin or anti-LC3-II antibody (diluted to 1 : 100 with Dako Antibody Diluent) and then processed by using Vectastain Elite ABC Peroxidase kit with Imm PACT DAB peroxidase substrate or exposed to chicken anti-goat IgG-FITC. Negative controls were performed by eliminating the primary or secondary antibody. Images were captured with a Nikon DXM 1200C camera using Nikon ACT-1C software.

### 2.8. Transmission Electron Microscopy

Transmission electron microscopy (TEM) using myotubes was performed for the visualization and quantitation of autophagic structures, as described previously [33].

### 2.9. Western Blot

Gastrocnemius muscles were homogenized in RIPA buffer plus Phosphatase Inhibitor and Complete Mini Protease Inhibitor (1 mg protein per 20 mL RIPA) and cell lysates were then centrifuged at 12,000 g for 10 min and the supernatants were collected. The protein concentrations were measured and 100 mg of protein was treated with SDS/DTT loading Buffer A prior to gel electrophoresis as described previously [34], using the following antibodies myostatin, Atg3, Atg7, Atg12, Beclin-1, LC3-I/II, p-PI3K, PI3K, p-Akt, Akt, p-FoxO3a, FoxO3a, p-FoxO1, MAFbx, MuRF1, PAMA4, Rpt5, Rpn11, MHC, and GAPDH. Images were captured and documented with a CCD system (image station 2000MM, Kodak, USA). The quantitative analysis of these images was performed using the Molecular Imaging Software Version 4.0 provided by Kodak 2000MM System.

### 2.10. Real Time Polymerase Chain Reaction

Gastrocnemius muscles were obtained from CKD and sham operated, pair-fed control rat. RNA was obtained using RNA easy; cDNAs were synthesized using iScript cDNA Synthesis Kit. Real time polymerase chain reaction was performed with CFX96 Real-Time PCR Detection System. The rat MAFbx primer sequences were forward 5′-GCTGGATTGGAAGAAGATG-3′, reverse 5′-TGAAAGTGAGACGGAGCAG-3′, MuRF-1 primer sequences were forward 5′-CGACCGAGTTCAGACTATCAT-3′, reverse 5′-TTGGCACTCAAGAGGAAGG-3′, GAPDH primer sequences were: forward 5′-TTCAACGGCACAGTCAAGG-3′, reverse 5′-CTCAGCACCAGCATCACC-3′.

### 2.11. NF-*κ*B Activity

C2C12 myoblasts were infected with an adenovirus expressing NF-*κ*B-luciferase. After differentiation into myotubes, the media were replaced and TNF-*α* were added. After harvesting, cellular luciferase activity was assayed according to Promega (Madison, WI).

### 2.12. Silencing Myostatin and Overexpression of Myostatin

C2C12 myoblasts were electroporated with either siRNAs or plasmid cDNAs using the Amaxa Nucleofector technology and protocol (Lonza). Myoblasts were transfected with 2 mg of plasmid myostatin or plasmid encoding GFP and then differentiated into myotube, and myotubes were placed in serum-free media and treated with 100 ng/mL TNF-*α* for 24 h. Alternatively, the myoblasts were transfected with 0.4 mg of myostatin siRNA or Control (scrambled) siRNA. The transfected cells were allowed to differentiate into myotubes and placed in serum-free medium before being treated with 100 ng/mL TNF-*α* for 24 h.

### 2.13. Statistics

Values are presented as means ± SD, and results were analyzed using Student's *t*-test when results from two experimental groups were compared or using analysis of variance when data from ≥3 groups were studied. For analysis of variance analyses, pairwise comparisons were made by the Student-Newman-Keuls test. Statistical significance was set at *P* < 0.05.

## 3. Results

### 3.1. Proteinuria and Renal Function

The serum albumin was in the normal range in the sham group, while they were decreased in the CKD group; moreover, significant differences were observed in between the 2 groups (*P* < 0.01). As for the BUN, SCr, and urinary protein levels, they were significantly increased in the CKD groups, as compared with that of the sham group (*P* < 0.01) (Table 1).

### 3.2. CKD Causes Muscle Atrophy and Accelerates Protein Degradation

**Figure 1:**
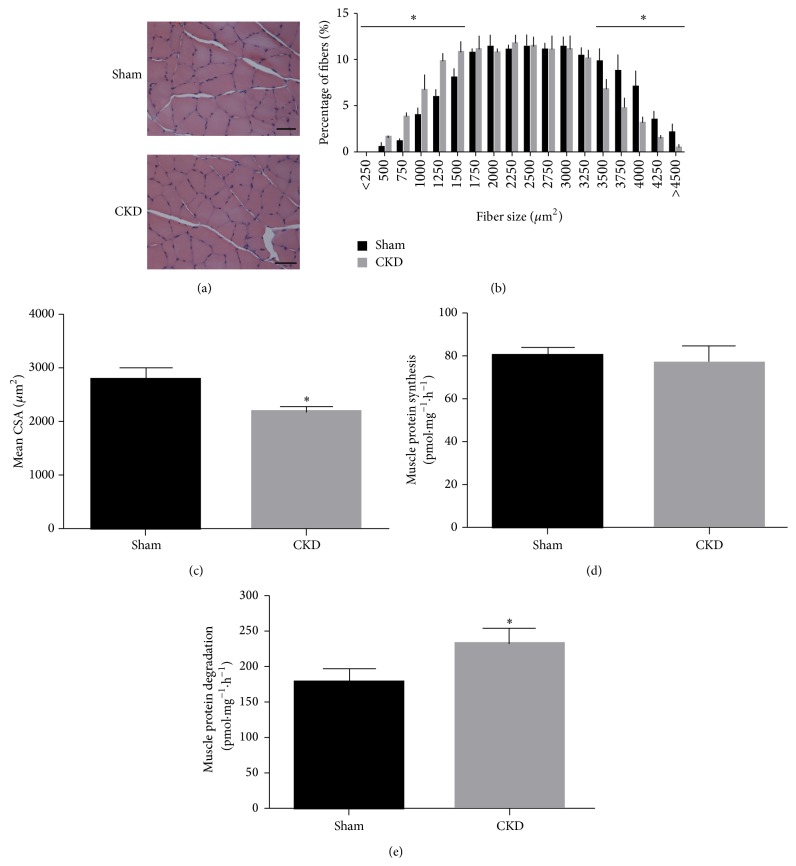
CKD decreases the cross-sectional area (CSA) of muscle fiber and accelerates protein degradation, but not affect protein synthesis in rat muscle. (a) Cross-sections of TA muscle stained with H&E. Scale bar = 50 *μ*m. (b) Muscle fiber frequency distribution of TA muscles. (c) Muscle fiber CSA (*μ*m^2^) of TA muscles. (d) Protein synthesis was measured from the rate of incorporation of l-[U-14C] phenylalanine into isolated, incubated soleus muscles. (e) Protein degradation was measured as the rate of tyrosine release from isolated soleus muscles. Values were described means, with SD represented by vertical bars. Significantly different (^*∗*^ *P* < 0.05, *n* = 3 independent experiments) from sham group.

The body weight was significantly lower in the CKD group as compared with the sham group (*P* < 0.01). The CKD group also displayed a significant reduction in the wet weight of gastrocnemius (Gastroc), tibialis (Sol), and anterior (TA) muscles (*P* < 0.01) when compared to the sham group. In addition, TA muscle dry weight (*P* < 0.01) and the ratio of TA muscle dry weight normalized to body weight (*P* < 0.05) were significantly decreased in the CKD group, as compared with that of the sham group (Table 2). The cross-sectional area (CSA) in measurement of the muscle fiber size was considered as the best indicator for muscle atrophy. Therefore, we measured the CSA of TA muscle (Figure 1(a)) and found a significant decrease in the average CSA of TA muscle in CKD group when compared with sham group (2843 ± 115 *μ*m^2^ versus 2163 ± 65 *μ*m^2^, *P* < 0.05) (Figure 1(c)). Moreover, there was a decrease in the percentage of large muscle fibers (>3500 *μ*m^2^), and an increase in the percentage of small fibers (<1500 *μ*m^2^) (Figure 1(b)). The results suggested there was a left shift in the myofiber distribution in CKD group versus sham group, confirming that CKD induced muscle atrophy. CKD induces muscle atrophy by impairing protein metabolism (2,30). We measured muscle protein synthesis and degradation and found a significant increase in protein degradation in CKD group as compared with sham group (243 ± 16 p mol mg^−1^ h^−1^ versus 182 ± 14 p mol mg^−1^ h^−1^, *P* < 0.05) (Figure 1(e)), but the protein synthesis was not statistically different between the CKD and sham groups (Figure 1(d)).

### 3.3. CKD Activates the Ubiquitin-Proteasome and Autophagy-Lysosome Systems in Skeletal Muscle

**Figure 2:**
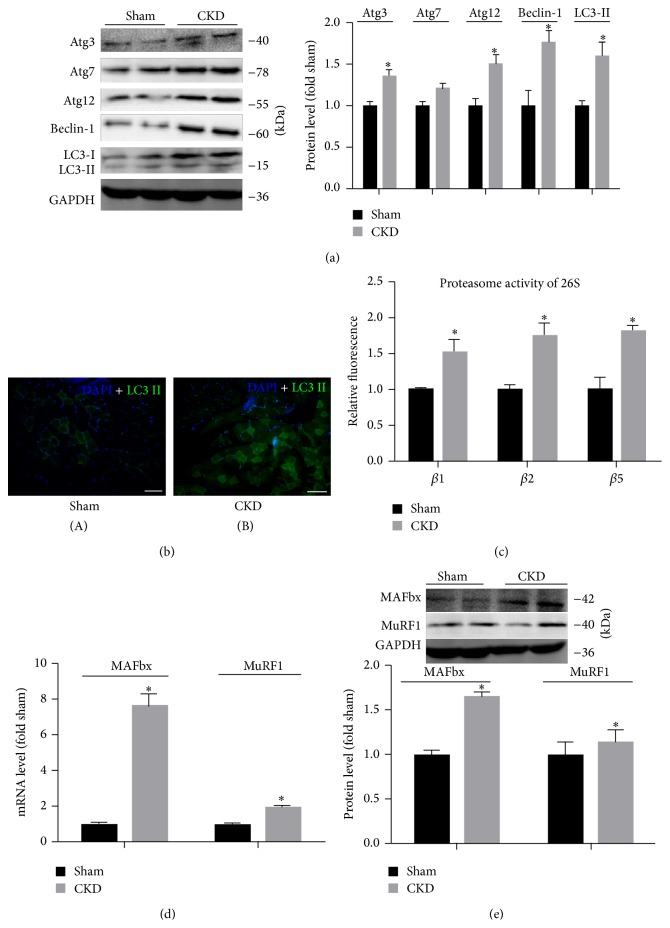
CKD upregulates the ubiquitin-proteasome and autophagy-lysosome systems in rat muscle. (a) Left: representative immunoblotting of Atg3, Atg7, Atg12, Beclin-1, LC3-I/II, and glyceraldehyde-3-phosphate-dehydrogenase (GAPDH). Right: The ratio of Atg3, Atg7, Atg12, Beclin-1, LC3-II, and GAPDH normalized to the sham group. (b) Representative fluorescent images of LC3-II. LC3-II was marked in green and nuclei were labeled by DAPI staining in blue. Scale bar = 50 *μ*m. (c) The caspase-like *β*1 activity, trypsin-like *β*2 activity, and chymotrypsin-like *β*5 activity of 26S in gastrocnemius muscle were measured after initiating the reaction with Z-LLE-AMC (*β*1), Boc-LSTR-AMC (*β*2), and Suc-LLVY-AMC (*β*5). (d) The expression of MAFbx and MuRF-1 mRNA was measured by RT-PCR and is presented as corrected for GAPDH and normalized to the sham group. (e) Upper: representative immunoblotting of MAFbx, MuRF1, and GAPDH. Lower: the ratio of MAFbx, MuRF1, and GAPDH normalized to the sham group. Values were described means, with SD represented by vertical bars. Significantly different (^*∗*^ *P* < 0.05, *n* = 3 independent experiments) from sham group.

The ubiquitin proteasome and autophagy-lysosomal systems are the two major proteolytic pathways involved in regulation of muscle wasting. To determine whether CKD activated the autophagosome pathway, the expression of autophagy and autophagy associated genes were examined. LC3-II levels were analyzed by immunofluorescence and immunoblotting, we found that CKD caused a marked elevation of LC3-II protein when compared with sham group (Figures 2(a)-2(b)). Moreover, there was a significant increase in the expression of autophagy related genes: Atg-3, Atg-12 and Beclin-1 expression in CKD group as compared to sham group, but it did not affect the expression of Atg-7 (Figure 2(a)). To analyze the ubiquitin-proteasome pathway on muscle atrophy, we evaluated the expression of proteasome activities and MAFbx and MuRF1. With respect to proteasome activities, the caspase-like *β*1 activity, trypsin-like *β*2 activity, and chymotrypsin-like *β*5 activity of 26S were stronger in the CKD group than those in the sham group (*P* < 0.05) (Figure 2(c)). There was a 7.7-fold increase of MAFbx and and 2.2-fold increase of MuRF1 in the muscles of CKD rat, as confirmed by RT-PCR (Figure 2(d)), whereas MAFbx protein increased in the muscles of CKD rat versus sham group, but the MuRF1 protein did not change (Figure 2(e)).

### 3.4. CKD Induces Myostatin Expression and Inhibts the PI3K/Akt/FoxO3a Signaling Pathway in Skeletal Muscle

**Figure 3:**
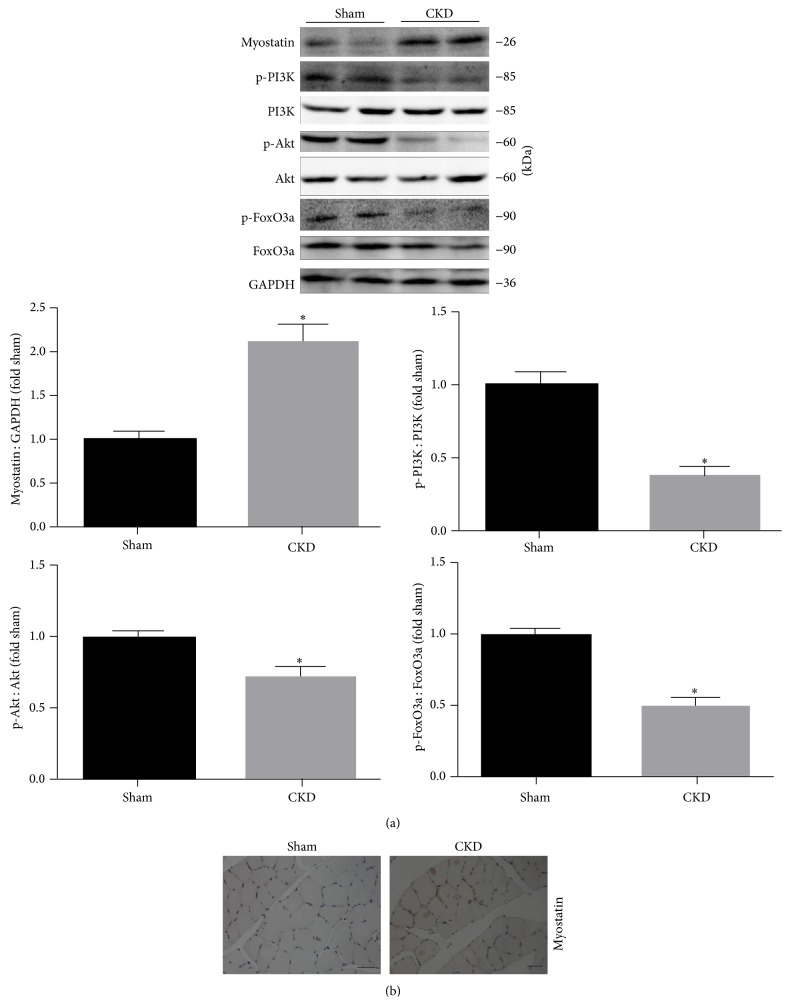
CKD upregulates myostatin expression and decreases the phosphorylation of PI3K, Akt, and FoxO3a in rat muscle. (a) Upper: representative immunoblotting of Myostatin, p-PI3K, PI3K, p-Akt, Akt, p-FoxO3a, FoxO3a, and GAPDH. Middle and Lower: The ratio of p-PI3K, p-Akt, and p-FoxO3a and PI3K, Akt, and FoxO3a normalized to the sham group. (b) Immunohistochemical staining for Myostatin in TA muscle. Scale bar = 50 *μ*m. Values were described means, with SD represented by vertical bars. Significantly different (^*∗*^ *P* < 0.05, *n* = 3 independent experiments) from sham group.

Evidence has been provided that Myostatin was increased in muscle wasting and the PI3K/Akt/FoxO3a pathway is also involved in myostatin-induced muscle atrophy. We examined Myostatin, p-PI3K, PI3K, p-Akt, Akt, p-FoxO3a, and FoxO3a protein levels by immunostaining and immunoblotting. The data indicate a 2.3-fold increase of myostain in CKD group when compared with sham group (*P* < 0.05; Figure 3(a)). By frozen sections of the TA muscle, we confirmed that myostain is principally present in muscle membranes and increases in CKD group (Figure 3(b)). Meanwhile, downward trend in PI3K, Akt, and FoxO3a phosphorylation in the CKD group was also observed as compared with the sham group (*P* < 0.05; Figure 3(a)).

### 3.5. In Differentiated Muscle Cells, TNF-*α* Stimulates Myostatin Expression Causing Myotube Atrophy

**Figure 4:**
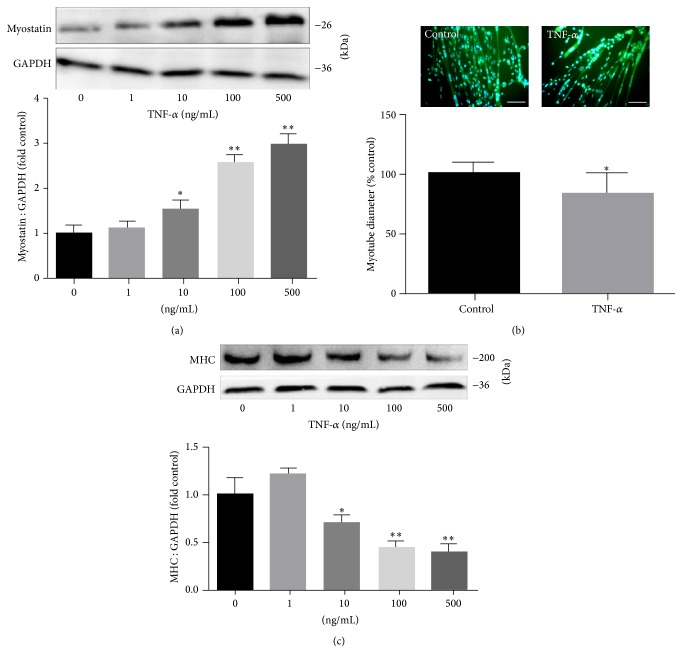
TNF-*α* induces the expression of myostatin and causes myotube atrophy in C2C12 myotubes. (a) Upper: representative immunoblotting of myostatin and GAPDH. Lower: The ratio of myostatin and GAPDH normalized to control after 24 hr of TNF-*α* treatment at the indicated concentrations. (b) Upper: representative fluorescent images of MHC. MHC was marked in green and nuclei were labeled by DAPI staining in blue. Lower: measurements of average myotube diameter after 24 hr of 100 ng/mL TNF-*α* treatment. Scale bar = 50 *μ*m. (c) Upper: representative immunoblotting of MHC and GAPDH. Lower: the ratio of MHC and GAPDH normalized to control after 24 hr of TNF-*α* treatment at the indicated concentrations. Values were described means, with SD represented by vertical bars. Significantly different (^*∗*^ *P* < 0.05, ^*∗∗*^ *P* < 0.01, *n* = 3 independent experiments) from control group.

To test our hypothesis, we carried out in vitro evaluation of the response to TNF-*α* by C2C12 myotubes that are known to express Myostatin. C2C12 myotubes were differentiated for 3 days, treated with a dose of TNF-*α* as indicated for 24 h-fixed, and assayed for changes in the expression of myostatin and MHC protein and myotube diameters. The data indicated the expression of myostatin and MHC increased in a dose-dependent fashion in response to TNF-*α* (Figures 4(a) and 4(c)). Immunofluorescence staining of the myotubes with an MHC-specific antibody revealed that exposure to 100 ng/mL TNF-*α* reduced the diameter of myotubes (*P* < 0.05; Figure 4(b)).

### 3.6. Myostatin Activates the Ubiquitin-Proteasome and Autophagy-Lysosome Systems via Dephosphorylation of PI3k/Akt/FoxO3a Signaling Pathway in Myotubes

**Figure 5:**
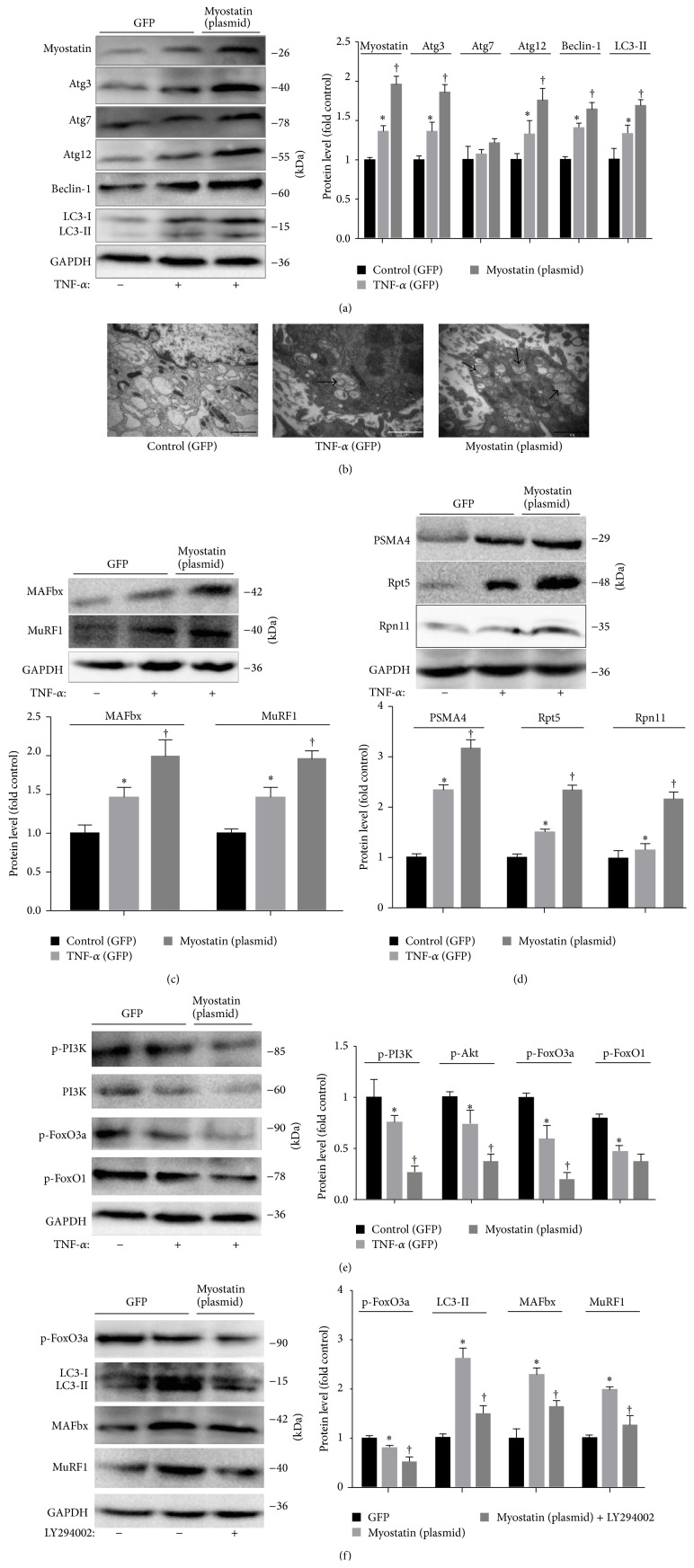
Overexpression of myostatin activates the ubiquitin-proteasome and autophagy-lysosome systems in myotubes via phosphorylation of PI3k/Akt/FoxO3a signaling pathway. C2C12 cells were transfected with myostatin plasmid versus plasmid expressing green fluorescent protein (GFP). After differentiating into myotubes, cells were treated with 100 ng/mL TNF-*α* for 24 h or LY294002 (50 *μ*M) for 1 h. (a) Left: representative immunoblotting of Atg3, Atg7, Atg12, Beclin-1, LC3-I/II, and GAPDH. Right: the ratio of Atg3, Atg7, Atg12, Beclin-1, LC3-II, and GAPDH normalized to control. (b) Representative electron micrograph of the autophagosomes. Autophagosomes were identified in the images as shown by arrows. Scale bar = 1 *μ*m. (c) Upper: representative immunoblotting of MAFbx, MuRF1 and GAPDH. Lower: The ratio of MAFbx, MuRF1, and GAPDH normalized to control. (d) Upper: representative immunoblotting of PAMA4, Rpt5, Rpn11, and GAPDH. Lower: the ratio of PAMA4, Rpt5, Rpn11, and GAPDH normalized to control. (e) Left: representative immunoblotting of p-PI3K, p-Akt, p-FoxO3a, p-FoxO1, and GAPDH. Right: the ratio of p-PI3K, p-Akt, p-FoxO3a, p-FoxO1, and GAPDH normalized to control. (f) Left: representative immunoblotting of p-FoxO3a, LC3-I/II, MAFbx, MuRF1, and GAPDH. Right: the ratio of p-FoxO3a, LC3-II, MAFbx, MuRF1, and GAPDH normalized to control. Values were described means, with SD represented by vertical bars. Significantly different (^*∗*^ *P* < 0.05) from control group, (^†^ *P* < 0.05, *n* = 3 independent experiments) from TNF-*α* or myostatin (plasmid) groups.

We examined whether the increase in CKD-stimulated myostatin affects the PI3k/Akt/FoxO3a signaling pathway in myotubes. C2C12 myoblasts were transfected with a plasmid that expresses myostatin or a green fluorescent protein (GFP)—expressing, control plasmid. After differentiating into myotubes, 100 ng/mL TNF-*α* was added for 24 h to activate the expression of myostatin (*P* < 0.05; Figure 5(a)). Transfection of myotubes with Myostatin plasmid increased myostatin expression by approximately 90% (Figure 5(a)). Transfection with the myostatin plasmid significantly increased the expression of LC3-II, Atg-3, Atg-12, Atg-7 and Beclin-1 protein versus myotubes with GFP transfection (*P* < 0.05; Figure 5(a)). To further confirm that myostatin induced the formation of autophagy, myotubes ultrastructure was analyzed using TEM. The results showed the formation of autophagosomes increased in myotubes transfected with myostatin plasmid (Figure 5(b)). Moreover, raising myostatin increased expression of the MAFbx and MuRF-1 (*P* < 0.05; Figure 5(c)), and caused a marked increase in PAMA4, Rpt5, Rpn11 expression (*P* < 0.05; Figure 5(d)). In addition, transfection with the myostatin plasmid significantly decreased p-PI3K, p-Akt, and p-FoxO3a versus myotubes with GFP transfection, which not affected p-FoxO1 expression (*P* < 0.05; Figure 5(e)). Our results showed that FoxO3a phosphorylation was significantly down-regulated by treatment with specific PI3K inhibitor LY294002. We found that inactivating FoxO3a prevent the myostatin-induced increase in MuRF1, MAFbx, and LC3-II protein expression (*P* < 0.05; Figure 5(f)).

### 3.7. Silencing Myostatin Inhibits Ubiquitin-Proteasome and Autophagy-Lysosome Systems in Myotubes Despite TNF-*α*

**Figure 6:**
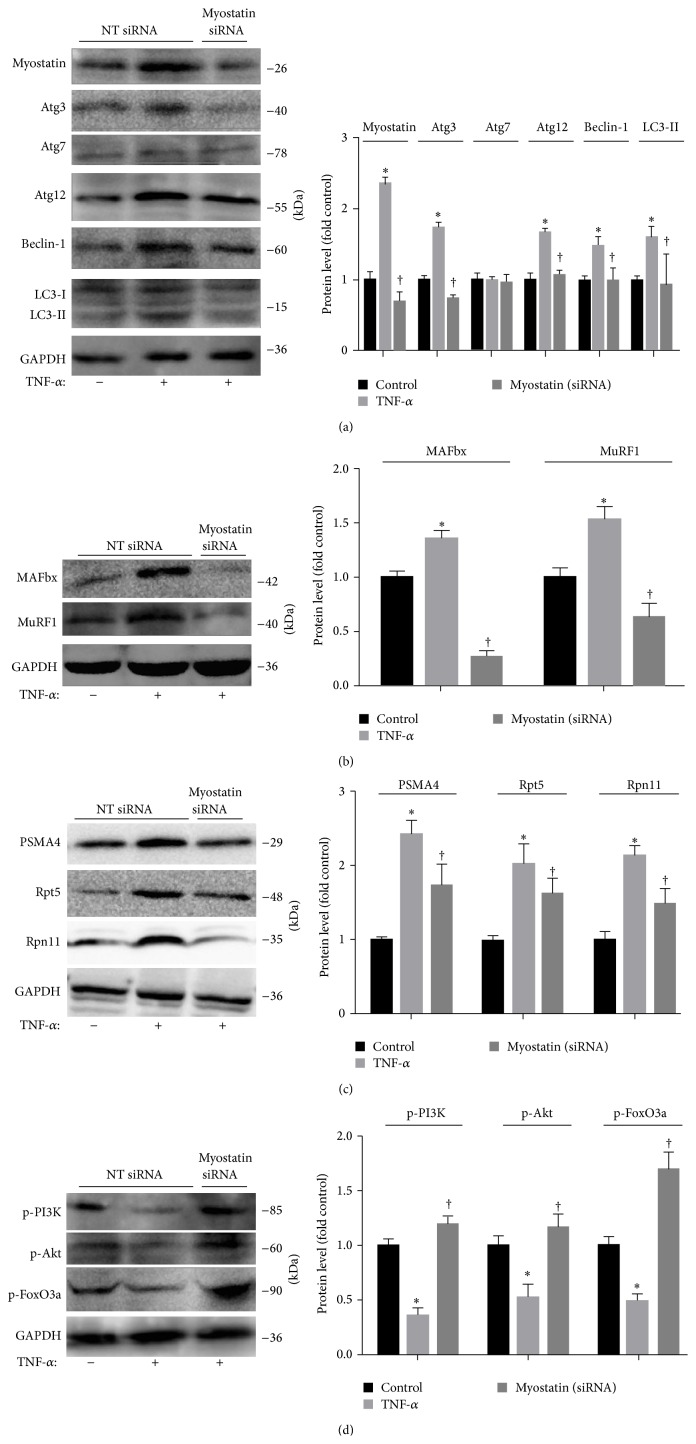
Suppression of myostatin inhibits the ubiquitin-proteasome and autophagy-lysosome systems in myotubes despite the presence of TNF-*α*. C2C12 cells were transfected with myostatin siRNA (myostatin) versus control scrambled siRNA (Control). After differentiating into myotubes, cells were treated with 100 ng/mL TNF-*α* for 24 h. (a) Left: representative immunoblotting of Atg3, Atg7, Atg12, Beclin-1, LC3-I/II, and GAPDH. Right: The ratio of Atg3, Atg7, Atg12, Beclin-1, LC3-II, and GAPDH normalized to control. (b) Left: representative immunoblotting of MAFbx, MuRF1, and GAPDH. Right: the ratio of MAFbx, MuRF1 and GAPDH normalized to control. (c) Left: representative immunoblotting of PAMA4, Rpt5, Rpn11, and GAPDH. Right: The ratio of PAMA4, Rpt5, Rpn11 and GAPDH normalized to control. (d) Left: representative immunoblotting of p-PI3K, p-Akt, p-FoxO3a, and GAPDH. Right: The ratio of p-PI3K, p-Akt, p-FoxO3a, and GAPDH normalized to control. Values were described means, with SD represented by vertical bars. Significantly different (^*∗*^ *P* < 0.05) from control group, (^†^ *P* < 0.05, *n* = 3 independent experiments) from TNF-*α* group.

As myostatin activates the ubiquitin-proteasome and autophagy-lysosome systems, its suppression should improve the ubiquitin-proteasome and autophagy-lysosome systems. To test this proposal, we examined whether silencing myostatin inhibits ubiquitin-proteasome and autophagy-lysosome systems in myotubes despite treatment with 100 ng/mL TNF-*α*. Myoblasts were treated with a myostatin small interfering RNA (siRNA) or a scrambled siRNA and stimulated to differentiate into myotubes. We found that silencing myostatin in myotubes exposed to TNF-*α* decreased LC3-II levels and autophagy related genes: Atg-3, Atg-12, and Beclin-1 expression (*P* < 0.05; Figure 6(a)). Moreover, silencing myostatin in myotubes exposed to TNF-*α* also decreased the protein expressions of MAFbx-1 and MuRF-1 (*P* < 0.05; Figure 6(b)), and caused a marked reduction in PAMA4, Rpt5, and Rpn11 expression (*P* < 0.05; Figure 6(c)). In addition, suppression of myostatin increased p-PI3K, p-Akt, and p-FoxO3a, even though TNF-*α* was present (*P* < 0.05; Figure 6(d)).

### 3.8. TNF-*α* Regulates Myostatin Expression via Nuclear Factor (NF)-*κ*B

**Figure 7:**
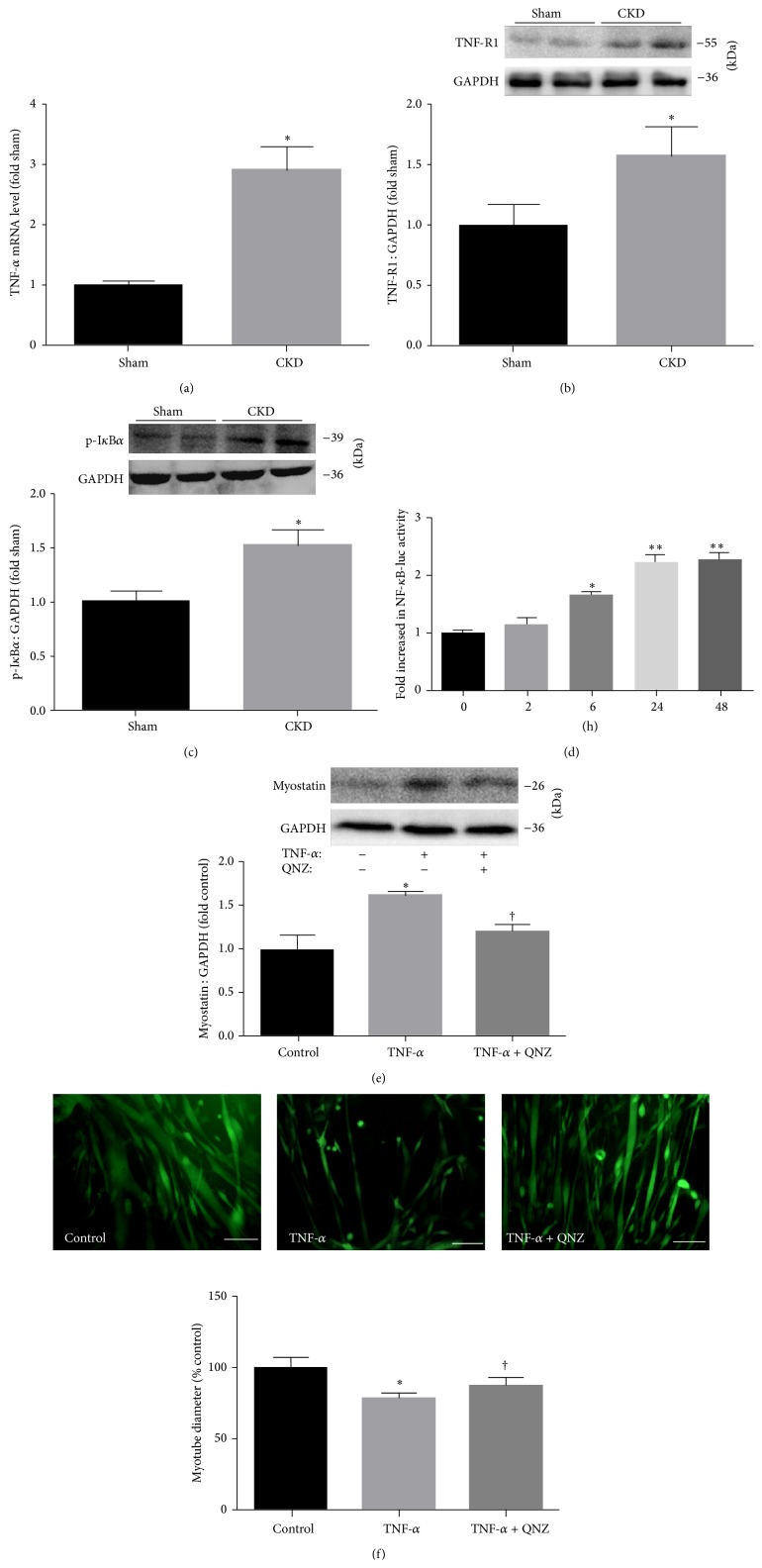
TNF-*α* regulates myostatin expression via nuclear factor (NF)-*κ*B. (a) The expression of TNF-*α* mRNA was measured by RT-PCR in rat muscle and is presented as corrected for GAPDH and normalized to Sham group. (b) The expression of TNF-R1 protein was measured by immunoblotting in rat muscle and is presented as corrected for GAPDH and normalized to Sham group. (c) The expression of p-IkBa protein was measured by immunoblotting in rat muscle and is presented as corrected for GAPDH and normalized to sham group. (d) C2C12 cells were infected with a NF-*κ*B-promoter luciferase construct. Following their differentiation, the myotubes were treated with 100 ng/mL TNF-*α*. Activation of the NF-*κ*B promoter at times listed was measured and the fold change over 0 h was quantified. (e) Myotubes were treated with 100 ng/mL TNF-*α* with or without the NF-*κ*B inhibitor (QNZ). Representative immunoblots of myostatin and GAPDH and the ratio of myostatin and GAPDH normalized to control. (f) Upper: representative fluorescent images of MHC. Lower: measurements of average myotube diameter after 24 hr of 100 ng/mL TNF-*α* treatment with or without the QNZ. Scale bar = 50 *μ*m. Values were described means, with SD represented by vertical bars. Significantly different (^*∗*^ *P* < 0.05, ^*∗∗*^ *P* < 0.01) from control group, (^†^ *P* < 0.05, *n* = 3 independent experiments) from TNF-*α* group.

As CKD rises circulating inflammatory cytokines, we hypothesized that they may affect myostatin expression and function through activation of NF-*κ*B. We found a significant increase in mRNA expression of TNF-*α* (*P* < 0.05; Figure 7(a)) and its receptor TNF-R1 protein (*P* < 0.05; Figure 7(b)) in muscle of CKD when compared to sham rats. We found a significant increase in the phosphorylation of I*κ*Ba in muscles of CKD rat (*P* < 0.05; Figure 7(c)), which would lead to I*κ*Ba degradation and translocation of NF-*κ*B into the nucleus and expression of target genes. Moreover, we infected C2C12 myoblasts with a NF-*κ*B promoter luciferase adenovirus. When they differentiated into myotubes, we found that TNF-*α* produced a significant increase in NF-*κ*B promoter activity at 6 or 24 or 48 h (*P* < 0.05; Figure 7(d)). To confirm NF-*κ*B involvement, we treated myotubes with a NF-*κ*B inhibitor (QNZ), and after 2 h added TNF-*α*. The TNF-*α* increased myostatin expression and its expression was suppressed by QNZ (*P* < 0.05; Figure 7(e)). In addition, muscle fiber diameter was decreased with TNF-*α* treatment, while being improved by QNZ (*P* < 0.05; Figure 7(f)). Thus, NF-*κ*B activation in muscle cells promotes myostatin expression.

## 4. Discussion

The results reported in this study provide several new observations on the direct and/or interactive effects of UPS, ALS, and myostatin in muscles of CKD. First, our data demonstrate that the UPS and ALS are activated. Second, myostatin protein expression is markedly upregulated. These findings are together associated with impaired muscle maintenance and subsequently muscle atrophy. Third, phosphorylated Akt is markedly decreased and closely associated with the loss of muscle mass caused by myostatin, which suggests that myostatin impaired Akt/FoxO3a pathway contributing to loss of myofibers. Finally, upregulation of myostatin is associated with expression of TNF-*α* gene and NF-*κ*B pathway, suggesting that microinflammatory changes occurring in muscle cells are signals for regulation of myostatin. Collectively, these results show the involvement of multiple catabolic signal transduction pathways, which may promote muscle wasting and impair muscle regeneration.

Previous studies have shown that activation of the proteolytic system in mammalian cells is transcriptionally regulated by several pathways, and a subset of target genes that has been identified in atrophying skeletal muscle, regardless of the catabolic illnesses [9, 10, 35, 36]. These genes are thought to regulate the loss of muscle components, and were thus designated atrophy-related genes or “atrogenes” [5, 36, 37]. These genes are involved in transcription related to the UPS pathway and ALS pathway. In muscle, the UPS is required to remove sarcomeric proteins in response to changes in muscle wasting. The rate-limiting step of the ubiquitination process, which affects subsequent proteasome-dependent degradation, is catalysed by the MAFbx and MuRF1, which are thought as both muscle-specific and upregulated proteins during muscle loss. Knockdown of MAFbx prevents muscle loss during fasting [38], whereas MuRF1 knockout mice are also resistant to muscle atrophy induced by dexamethasone [39]. The evidence in the present study indicates that the expression of MAFbx and MuRF1 mRNA was increased in the muscles of CKD rat, and the increase of MAFbx protein level and upregulation of proteasome activity of 26S were associated with increased muscle protein degradation in CKD-induced muscle atrophy. In addition, we found increased MAFbx, MuRF1 protein and proteasome subunits in TNF-*α*-induced myotubes atrophy in C2C12 myotubes correlated with downregulation of MHC protein. These data strongly suggest that the UPS is activated in atrophying muscles in CKD.

There are several lines of evidence suggesting that the ALS is activated in muscle cells during catabolic conditions [40–42]. Mizushima et al. [43] generated transgenic mice expressing LC3 fused with GFP, which is the mammalian homolog of Atg8 gene and is critical for membrane commitment and growth to engulf organelles, cytoplasm, glycogen, and protein aggregates. Morphological analyses documented the activation of the autophagy system in skeletal muscle during fasting [43]. Indeed, muscle cell culture confirmed that the autophagy-lysosome system is the major proteolytic pathway implicated in nutrient-dependent proteolysis [44]. Further experiments lend insight into the signaling pathways involved and identified an mTOR-independent but PI3K-beclin-dependent control of the autophagic system in myotubes [42]. Furthermore, electron microscopic and biochemical studies have also shown that autophagy is activated in denervation atrophy [45, 46]. Our evidence in this study demonstrate that the genes related to autophagy including Atg-3, Atg-12, LC3-II and Beclin-1 were increased in muscles of CKD rats, as well as in C2C12 myotubes treated with TNF-*α*, which suggests that the activation of ALS mediates the degradation of myofibrillar and nonmyofibrillar proteins. Consistent with the previous reports [47], our results demonstrate that TNF-*α* resulted in the increase of the expression of both the MAFbx and LC3-II genes involved in protein degradation in myotubes. Our results were corresponds to a previous study that the mRNA expressions of LC3, GabarapI1, and Cathepsin L were upregulated in the skeletal muscle of uremia rats induced by subtotal nephrectomy [35] and by type 2 diabetic nephropathy [48]. These findings reveal that ALS pathway was activated in muscle atrophy induced by TNF-*α* or CKD.

Myostatin is a well-known negative regulator of muscle mass, and myostatin expression is increased in a number of diseases where muscle wasting is also observed [6, 17, 18]. In the study, we examined whether activation of the myostatin contributed to upregulation of the UPS pathway. First, we showed that the UPS was upregulated associated with activation of myostatin in muscles of CKD rat. Second, overexpression of myostatin in myotubes resulted in upregulation of the UPS. Conversely, silencing myostatin in myotubes promoted elevation of the UPS despite the presence of TNF-*α*. Previously evidence reported that MAFbx increasingly interacts with MyoD upon recombinant human myostatin treatment during the myostatin-induced skeletal muscle wasting [49], which is consistent with our results. The data reported in this paper provide novel evidence that myostatin is an activator of UPS in muscles of CKD. We further showed that myostatin overexpression increases the expression of components of the autophagy pathway. Another report showed that myostatin induced increase of LC3-II expression and turnover, as well as autophagosome formation, which is also consistent with our data [50]. In addition, silencing myostatin promoted increase of the ALS in myotubes despite the presence of TNF-*α*. Collectively, our data therefore raise the possibility that myostatin enhanced autophagy and proteasomal degradation together contribute to the loss of muscle mass.

As showed in previous observations, expression of myostatin was associated with up-regulation of inflammatory cytokines, suggesting its dependency on microinflammatory changes occurring within the skeletal muscle [6, 51]. Our results demonstrate that CKD induces increase of TNF-*α* mRNA and TNF-R1 protein associated with activation of NF-*κ*B that stimulates a twofold to threefold increase in the expression of myostatin, leading to muscle protein wasting. We also demonstrated that TNF-*α* treatment increased myostatin expression via a NF-*κ*B dependent pathway in C2C12 myotubes, and inhibition of NF-*κ*B activity suppresses myostatin and improves muscle wasting. Our results were consistent with a previous study that TNF-*α* increased myostatin expression via a NF-*κ*B-dependent pathway; inhibition of myostatin suppresses systemic inflammation and muscle atrophy in mice with CKD [51]. Thus, an increase in TNF-*α* will stimulate NF-*κ*B associated with upregulation of myostatin contribution to muscle wasting.

The upregulation of both the UPS and ALS are normally blocked by Akt through negative regulation of FoxO transcription factors [16, 37, 52, 53]. Activation of FoxO transcription factors is responsible for the atrophy induced by denervation or fasting, and atrophy of muscles and myotubes was markedly caused by activation of FoxO3a, a transcription factor known to control protein degradation via transcriptional activation of both UPS and ALS components [16]. In our study, the phosphorylation of Akt was drastically decreased and was associated with a major dephosphorylation of its downstream target FoxO3a in myotubes. In culture experiments, overexpression of myostatin decreases the phosphorylation state of Akt and the dephosphorylates of its downstream target FoxO3a, and silencing myostatin increases the phosphorylation state of Akt and phosphorylates of its downstream target FoxO3a in C2C12 myotubes despite the presence of TNF-*α*. In addition, our results show that inhibition of phosphoinositol-3 kinase (PI3K)/AKT signaling with LY294002 reduces FoxO3a phosphorylation and prevents the myostatin-induced increased in MuRF1 and MAFbx and in autophagy protein expression. Our previous findings showed that TNF-*α*-induced muscle atrophy via regulation of Akt/FoxO1/3a patyway in C2C12 myotubes [27]. TNF-*α* induction of MAFbx mRNA depends on Foxo4 expression but not AKT-Foxo1/3 signaling [54]. There is a disagreement between the previous evidence and the observations reported here. On the whole, our results indicate that myostatin enhanced autophagy and proteasomal degradation together contribute to the loss of muscle mass through the Akt/FoxO3a signaling pathway.

In conclusion, our results emphasize that stimulation of myostatin contributes to a serial of pathophysiological consequences: myostatin enhances protein degradation in muscles and TNF-*α* cannot stimulate protein degradation any more in muscle cells when myostatin was silenced. In contrast, myostatin overexpression was shown to stimulate the activation of the UPS and ALS via the Akt/FoxO3a pathway contributing to the increase of protein degradation in muscle cells. We also demonstrate that CKD upregulates myosatin expression in muscle and that was associated with increased formation of autophagosome and expression of ubiquitin ligase in rat. In conclusion, our findings suggest that targeting myostatin may prove to be a therapeutic modality, owing to improvement of the UPS and ALP and prevention of muscle wasting in CKD.

## Figures and Tables

**Table 1:** Biochemical data evaluating kidney function.

	Sham	CKD

BUN (mmol/L)	8.51 ± 1.59	16.53 ± 4.40^**^
Creatinine (mmol/L)	36.30 ± 5.93	99.38 ± 7.37^**^
Serum albumin (g/L)	40.67 ± 3.22	33.48 ± 3.82^**^
Urinary protein (mg/24 h)	5.56 ± 1.43	31.41 ± 10.82^**^

BUN: blood urea nitrogen; CKD: chronic kidney disease. Serum BUN, creatinine, serum albumin, and urinary protein were evaluated in CKD versus sham-operated control rat. (^*∗∗*^ *P* < 0.01 versus sham).

**Table 2:** Body weight, TA, Gastroc, and Sol muscle weight data.

	Sham	CKD

Body weight (g)	604.8 ± 21.5	590.9 ± 11.8^**^
Gastroc MWW (mg)	2900.9 ± 105.3	2389.8 ± 89.5^**^
Sol MWW (mg)	578.9 ± 32.1	403.7 ± 35.1^**^
TA MWW (mg)	982.3 ± 53.5	767.8 ± 56.4^**^
TA MDW (mg)	276.0 ± 11.0	212.2 ± 15.4^**^
TA MDW/BW (mg/g^−1^)	0.46 ± 0.02	0.38 ± 0.05^*^

TA: tibialis anterior; Gastroc: gastrocnemius; Sol: soleus; MWW: muscle wet weight; MDW: muscle dry weight; BW: body weight. Body weight, muscle wet weight, muscle dry weight, and MDW/BW were evaluated in CKD versus sham-operated control rat. (^*∗*^ *P* < 0.05 and ^*∗∗*^ *P* < 0.01 versus sham).
